# Chronic life stressors and pain incidence in adults 50 and older: a longitudinal analysis with implications for pain prevention and public health promotion

**DOI:** 10.3389/fpubh.2026.1905906

**Published:** 2026-08-04

**Authors:** Antoinette L. Spector, Dina Flores, David Aaby, Patricia Coe

**Affiliations:** 1 Department of Physical Therapy and Human Movement Science, Feinberg School of Medicine, Northwestern University, Chicago, IL, United States; 2 Division of Biostatistics, Department of Preventive Medicine, Feinberg School of Medicine, Northwestern University, Chicago, IL, United States; 3 National University of Health Sciences, Lombard, IL, United States

**Keywords:** aging, biopsychosocial model, chronic life stressors, chronic pain, health promotion, non-pharmacological interventions, pain prevention, population health

## Abstract

**Background:**

Chronic pain represents a pressing public health problem affecting more than one in five American adults. Yet, primary prevention efforts aimed at stopping pain before it begins remain substantially underutilized. Chronic life stressors (CLS)—including financial strain, personal health problems, and caregiving burdens—are upstream, modifiable psychosocial risk factors that may drive pain incidence in aging populations. Identifying and addressing these stressors at the population level offers a promising, non-pharmacological pathway for preventing new-onset pain.

**Methods:**

We used data from the 2018 and 2020 Health and Retirement Study (HRS), a nationally representative longitudinal study of U.S. adults aged 50 and older (*N* = 14,133). Among 2,622 individuals reporting no pain at baseline (2018), we examined the association between eight CLS domains and pain incidence in 2020 using unadjusted and adjusted logistic regression models, adjusting for gender, race/ethnicity, education, marital status, employment status, and self-rated health.

**Results:**

In unadjusted models, moderate-to-severe self-health problems (OR = 2.13, 95% CI: 1.63–2.79, *p* < 0.001), physical/mental health problems in a spouse or child (OR = 1.46, 95% CI: 1.11–1.93, *p* = 0.007), and financial strain (OR = 1.62, 95% CI: 1.16–2.27, *p* = 0.005) were significantly associated with pain incidence. In fully adjusted models, moderate-to-severe self-health problems remained significant (OR = 1.62, 95% CI: 1.20–2.19, *p* = 0.002), and cumulative CLS exposure predicted pain incidence (OR = 1.13, 95% CI: 1.05–1.23, *p* = 0.002), with each additional stressor increasing pain odds by 13%.

**Conclusion:**

These findings provide longitudinal evidence that life stressors are upstream, modifiable determinants of new-onset pain among adults 50 and older, as well as viable targets for public health prevention programs. Population-level implementation of social needs screening, combined with clinical and community-based support, offers a proactive strategy to prevent the transition from no pain to chronic pain. These results support a paradigm shift from reactive pain management toward salutogenic, prevention-oriented public health approaches that promote healthy aging and reduce downstream pharmaceutical dependence.

## 1 Introduction

Findings from the Centers for Disease Control and Prevention (CDC) showed a chronic pain prevalence of more than 20% among Americans in 2021, with some individuals managing pain symptoms so severe it reduced their participation in life activities (1). In contrast to pain prevalence—the proportion of individuals in a population who have pain at a given point or over a specified period—pain incidence is the number or rate of new cases of pain that develop in a previously pain-free population during a defined time period. Studying pain incidence is crucial for public health and clinical care alike because it identifies how often pain first emerges, which risk factors predict the transition from no pain to chronic pain, and where prevention and early intervention efforts should be targeted (2).

Understanding how chronic life stressors (CLS) affect pain incidence, particularly in later life, is an understudied but critical area in the primary prevention of chronic pain (3). Social determinants of health (SDOH)—such as food and housing insecurity, inability to access quality healthcare, financial instability, and inadequate social support—can all lead to experiencing CLS (4) and, consequently, to higher incidence of pain later in life. The biopsychosocial model of pain is a widely accepted framework that emphasizes biological, psychological, and social contributions to the pain experience (5). Yet social determinants of pain remain understudied, despite evidence that social and environmental factors account for as much as 70% of health outcomes (6). Adults over 50 are more likely to suffer from musculoskeletal conditions, have multiple chronic health conditions, and take multiple medications (7–9). They also more frequently carry care responsibilities for both children and aging parents (10), exposing this population to increased physical, mental, and financial life stressors that may activate the stress response and manifest physically as higher pain incidence.

Empirical research supports the association between CLS and pain. A meta-analysis of 45 studies (*N* > 175,000) found that individuals with low socioeconomic status had a 32% increased risk of chronic pain compared to those with high SES (11). A cross-sectional analysis of the 2018 Health and Retirement Study (HRS) found that middle-aged and older adults with four or more chronic life stressors had over twice the odds of experiencing any chronic pain and a four-fold increase in the odds of high-impact chronic pain (12). The present study extends this work by using a longitudinal design to examine whether baseline CLS predict new-onset pain incidence among initially pain-free adults over 50—a design that enables temporal inference about the direction of the CLS-pain relationship and that provides evidence directly relevant to primary prevention.

Many adults who are pain-free in midlife go on to develop new-onset pain as they age. Among pain-free older adults in Sweden, the annual incidence of chronic pain has been estimated at approximately 5%, and longitudinal data indicate that once chronic pain develops and persists, the likelihood of recovery diminishes with older age (2, 13). This trajectory—from no pain to persistent, chronic pain—represents a high-priority but underutilized target for public health prevention. Research on pain incidence in pain-free populations is fundamentally distinct from research on pain management in those already affected: rather than reducing symptoms or slowing progression, the goal is to identify modifiable risk factors that can be addressed before pain ever develops. The present study is grounded in this prevention-oriented framework, examining which chronic life stressors predict the transition to new-onset pain over a 2-year period among adults over 50 who were pain-free at baseline.

This study aims to explore the relationship between baseline CLS in 2018 and pain incidence in 2020, with focus on the cumulative impact of CLS among initially pain-free individuals over 50. Both the combined effect of multiple chronic stressors and the influence of individual stressors on pain progression will be assessed. Foregrounding the public health prevention imperative, we hypothesize that CLS are associated with an increase in pain incidence, and that there will be a cumulative dose–response effect for individuals experiencing multiple CLS. In addressing these hypotheses, this study provides empirical grounding for population-level prevention strategies aimed at older adults who are currently pain-free but at elevated risk.

## 2 Materials and methods

### 2.1 Data source

We analyzed data from the 2018 and 2020 University of Michigan Health and Retirement Study (HRS), an ongoing nationally representative, longitudinal study of individuals over 50 years of age in the United States (14). The HRS surveys over 20,000 participants every 2 years, since 1992, and provides information across many aspects of older adults’ lives, such as healthcare, finances, employment, and wellbeing (15, 16). We only included participants who responded to both the 2018 and 2020 surveys and completed questions related to physical health and CLS. The HRS dataset used for this study includes 14,133 adults over 50 who completed questionnaires in both 2018 and 2020 to examine the relationship between CLS and pain incidence for individuals who report no pain at baseline.

### 2.2 Outcomes and study measures

The primary outcome is pain incidence in 2020 for individuals who reported no pain in 2018. The HRS captures pain through self-report. The survey first asks all respondents: “Are you often troubled with pain?” Participants who previously responded “No” to this question in 2018 and subsequently answered “Yes” to this question in 2020, were coded as having pain, an approach that has previously been used to estimate pain prevalence among HRS participants (12, 17, 18). We examined eight specific life stressors from the 2018 survey. Chronic life stressors were operationalized in the HRS as events that were “current and ongoing problems that had lasted 12 months or longer.” A total of eight items were rated on a 4-point scale ranging from 1, “no, not a problem” to 4, “yes, very upsetting.” Each item was transformed into a binary variable (yes/no) based on if they indicated the following problems were “moderately upsetting” or “very upsetting” (19, 20): self-health problems, physical/emotional problems in a spouse or child, problems with alcohol or drug use in a family member, difficulties at work, financial strain, housing problems, problems in a close relationship, and helping at least one sick, limited, or frail family member or friend on a regular basis. We also created a sum total of all moderate-to-severe CLS, ranging from 0 to 8.

### 2.3 Data analysis

All analyses were conducted using R version 4.3.1. Descriptive statistics summarize demographic information and CLS by pain incidence in 2018. Among participants who felt no pain in 2018, we used logistic regression to examine the relationship between each CLS and pain incidence in 2020. We also used logistic regression to examine the association between pain incidence in 2020 and the total number of CLS. We presented unadjusted odds ratios (OR) and 95% confidence intervals (CIs) as well as ORs and 95% CIs adjusted for gender (female, male), race/ethnicity (Hispanic, Non-Hispanic Black, Non-Hispanic White, Other Non-Hispanic), education (less than high school, high school diploma/GED, some college, 4-year degree or above), marital status (married/partnered, widowed, separated/divorced, never married), employment status (employed, unemployed), and self-rated health (excellent, very good, good, fair, poor). The unadjusted analytic sample included *N* = 2,622 participants who reported no pain at baseline, had a valid response to the 2020 pain item, had a valid survey weight, and completed all chronic life stressor questions. Adjusted models included *N* = 2,603 participants; the reduction of 19 participants was due to item-level missing data in race/ethnicity, education, and employment status. All models were weighted to account for survey design and to reflect the US population. We used Taylor series linearization to estimate standard errors. A *p*-value less than 0.05 (*p* < 0.05) was used to define statistical significance.

## 3 Results

### 3.1 Sample characteristics

Table 1 summarizes the characteristics of the target sample. Of the 14,133 participants who received the 2018 HRS survey, 2,622 participants who reported no chronic pain in 2018 were included in the analytic sample. Of the 2,622 participants who reported no pain in 2018, 18% (*N* = 481) reported having pain symptoms in 2020. Our analytic sample (*N* = 2,662) consists of individuals with a mean age of 68.0, with a higher proportion identifying as female (56%), Non-Hispanic White (67%), who completed a 4-year college or above (36%), and currently being unemployed (55%). In addition, the majority self-rated their health as “Very good” (41%) or “Good” (34%).

**Table 1:** Characteristics of participants reporting no chronic pain at baseline (*N* = 2,662).

Characteristic	*n* (%)
Age (mean, SD)	68.0 (9.7)
Gender	
Male	1,155 (44%)
Female	1,467 (56%)
Race/ethnicity	
Hispanic/Latino	313 (12%)
Non-Hispanic Black	437 (17%)
Non-Hispanic White	1,765 (67%)
Other Non-Hispanic	101 (3.9%)
Unknown	6
Education	
Less than high school	305 (12%)
High school/GED	716 (27%)
Some college	653 (25%)
4-Year college or above	940 (36%)
Unknown	8
Employment status	
Employed	1,187 (45%)
Unemployed	1,433 (55%)
Unknown	2
Self-rated health	
Excellent	339 (13%)
Very good	1,068 (41%)
Good	894 (34%)
Fair	288 (11%)
Poor	32 (1.2%)
Unknown	1
Pain in 2020	
Yes	481 (18%)
No	2,141 (82%)

SD, standard deviation. Unknown/missing values excluded from percentages.

### 3.2 Unadjusted models

Table 2 displays ORs and 95% CIs describing the unadjusted associations between chronic pain incidence in 2020 with each CLS. Univariate models were performed on a total of 2,622 individuals after excluding all missingness. Individuals with moderate-to-severe self-health problems in 2018 had over twice the odds of chronic pain 2 years later compared to those without (OR = 2.13, 95% CI: 1.63–2.79, *p* < 0.001). Reporting chronic pain in 2020 was also associated with moderate-to-severe physical or mental problems in a spouse or child (OR = 1.46, 95% CI: 1.11–1.93, *p* = 0.007), as well as moderate-to-severe financial strain (OR = 1.62, 95% CI: 1.16–2.27, *p* = 0.005). Each additional stressor raised the likelihood of chronic pain incidence by 18% (OR = 1.18, 95% CI: 1.09–1.27, *p* < 0.001).

**Table 2:** Unadjusted logistic regression: Chronic life stressors and pain incidence in 2020 (*N* = 2,622).

Chronic life stressor	OR	95% CI	*p*-value
Self-health problems	2.13	1.63–2.79	<0.001^*^
Physical/mental health problems in a spouse or child	1.46	1.11–1.93	0.007^*^
Financial strain	1.62	1.16–2.27	0.005^*^
Alcohol/drug problems in family member	1.19	0.82–1.73	0.370
Difficulties at work	0.99	0.63–1.55	>0.900
Housing problems	1.59	1.00–2.54	0.051
Problems in a close relationship	1.31	0.90–1.89	0.200
Caregiving for sick/frail family or friend	1.26	0.88–1.80	0.200
Cumulative CLS (per additional stressor)	1.18	1.09–1.27	<0.001^*^

OR, odds ratio; CI, confidence interval. ^*^Statistically significant at *p* < 0.05.

### 3.3 Adjusted models

Table 3 displays ORs and 95% CIs describing the association between pain incidence in 2020 with each CLS, after adjusting for demographic and health characteristics. Multivariate logistic regression models were performed on a total of 2,603 individuals after excluding all missingness. Even after adjusting for demographic and health characteristics, those experiencing moderate-to-severe self-health problems had 1.62 times the odds of reporting pain compared to those who did not experience such issues (OR = 1.62, 95% CI: 1.20–2.19, *p* = 0.002). For each additional stressor experienced, the odds of pain incidence increased by 13% (OR = 1.13, 95% CI: 1.05–1.23, *p* = 0.002). After adjusting for demographic and health characteristics, there were no longer associations between pain in 2020 and moderate-to-severe physical/mental health problems in a spouse or children and moderate-to-severe financial strain.

**Table 3:** Adjusted logistic regression: moderate-to-severe chronic life stressors and pain incidence in 2020 (*N* = 2,603), adjusted for gender, race/ethnicity, education, marital status, employment status, and self-rated health.

Chronic life stressor	OR	95% CI	*p*-value
Self-health problems	1.62	1.20–2.19	0.002^*^
Physical/mental health problems in a spouse or child	1.30	0.97–1.72	0.075
Financial strain	1.39	0.98–1.98	0.067
Alcohol/drug problems in family member	1.08	0.74–1.59	0.700
Difficulties at work	1.14	0.71–1.84	0.600
Housing problems	1.43	0.86–2.36	0.200
Problems in a close relationship	1.41	0.97–2.05	0.074
Caregiving for sick/frail family or friend	1.25	0.86–1.81	0.200
Cumulative CLS (per additional stressor)	1.13	1.05–1.23	0.002^*^

OR, odds ratio; CI, confidence interval. ^*^Statistically significant at *p* < 0.05.

We also performed adjusted models excluding self-rated health as a covariate to evaluate the degree to which its inclusion might cause an underestimation of the true effect of CLS on pain incidence. Results were similar, but we note two significant differences. When removing self-rated health, moderate-to-severe physical/mental health problems in a spouse or children and moderate-to-severe financial strain were also significantly associated with pain.

## 4 Discussion

This study provides longitudinal evidence that CLS—upstream, modifiable psychosocial risk factors—are meaningfully associated with new-onset pain among adults 50 and older who were initially pain-free. By tracking HRS participants from 2018 to 2020, this analysis moves beyond cross-sectional documentation of pain prevalence to address a central question for public health prevention: which modifiable stressors predict the transition from no pain to chronic pain, and where should population-level prevention programs be directed? Study results are consistent with prior work on pain prevalence (12), confirming that personal health concerns are strong predictors of pain incidence and that the cumulative burden of CLS exerts a statistically and clinically meaningful effect. The current study’s findings also align with prior work documenting associations between CLS and pain outcomes. Brennan (21) found that stressors—including financial strain, health problems, and interpersonal difficulties—were significantly elevated among older adults with pain compared to those without pain (21). Taken together, this body of evidence highlights the role of modifiable psychosocial stressors as upstream determinants of pain, and the present study extends it by examining CLS prospectively in a pain-free sample—enabling temporal inference about which stressors predict new-onset pain before it develops. Critically, the dose–response relationship identified here—a 13% increase in pain odds per additional stressor in the adjusted model—underscores the public health imperative to reduce the psychosocial stressor burden across the older adult population, not only among individuals already receiving pain treatment.

### 4.1 Social needs screening as a public health prevention strategy

Our findings provide empirical support for integrating social needs screening into clinical and community-based encounters with older adults, especially those who are currently pain-free but carry elevated psychosocial stressor burdens. A social need is described as the need of an individual as a result of SDOH. Thus, investment in addressing social needs through social services (e.g., housing, financial resources, food access), care coordination, and community outreach may reduce exposure to the chronic life stressors that precipitate pain incidence (22). The rationale for screening is threefold: (1) CLS are prevalent and often unrecognized in routine clinical and public health assessments; (2) validated, brief screening instruments already exist and are feasible for implementation across diverse care settings; and (3) screening results can directly inform referral pathways, tailored multidisciplinary care plans, and community-level resource allocation.

The current study’s findings support the use of comprehensive screening instruments that capture multiple SDOH domains simultaneously, rather than single-item assessments. The AHC HRSN Screening Tool (10 items covering five core plus eight supplemental domains), PRAPARE (12 core questions across eight domains), and Health Leads Screening Toolkit are all designed to identify patients with multiple co-occurring social needs (23). Of the 25 SDOH screening tools identified in a recent systematic review, 18 studies proposed specific interventions to address the social needs identified through screening, including referral to social workers, community-based agencies, and web-based resource platforms that connect patients with local services (23). Community health centers, federally qualified health centers (FQHCs), and primary care settings are particularly well-positioned as implementation sites for population-level social needs screening given their reach into underserved communities with high stressor burden.

### 4.2 Public health and policy implications

The findings of this study carry broad implications for public health policy and for the structural conditions that determine pain risk in aging populations. Most fundamentally, they support an upstream public health approach to chronic pain by identifying modifiable psychosocial stressors that predict pain onset 2 years before it occurs. This study provides evidence that chronic pain is, in part, a preventable condition—one whose incidence can potentially be reduced through population-level strategies that address the stressor burden of older adults.

The alignment with Healthy People 2030 is direct. The Healthy People 2030 framework includes explicit goals related to reducing chronic pain prevalence, improving pain management, and addressing SDOH as drivers of health equity (24). The dose–response relationship documented here—each additional stressor conferring 13% greater pain odds—provides quantitative support for the Healthy People 2030 rationale. Reducing the number of co-occurring stressors in the older adult population, even marginally, could translate into meaningful reductions in chronic pain incidence at the population level. Public health campaigns and community health initiatives that reduce financial strain, alleviate caregiving burden, and improve access to mental health resources are therefore not merely social welfare priorities but evidence-based chronic pain prevention strategies.

### 4.3 Strengths

This study used a longitudinal design, allowing participants to be tracked over multiple years and thus the ability to assess temporal relationships between baseline life stressors and pain incidence. Eight specific domains of life stress (e.g., financial strain, housing problems, relationship problems) were examined, allowing identification of life stressors that are most predictive of pain. Demographic and health-related variables were adjusted in models, reducing potential confounding and increasing confidence in observed associations. The nationally representative HRS sample enhances the generalizability of findings to the broader U.S. adult 50 and older population.

### 4.4 Limitations

Several limitations of this study should be considered when interpreting the findings. First, the HRS sample is restricted to adults aged 50 and older residing in the United States, which may limit generalizability to younger populations or to adults in countries with different healthcare systems, social safety nets, or cultural contexts surrounding stress and pain. Second, although eight specific life stressor domains were evaluated, this set may not encapsulate the full breadth of chronic life stress or adequately capture an individual’s subjective experience of stressor severity and meaning. Third, while this study adjusted for several demographic and health confounders, unmeasured factors—including sedentary behavior, diet and nutrition, genetic predispositions, sleep quality, and stress resilience—could influence the relationship between CLS and pain incidence and represent important targets for future analyses. Fourth, while the current study presents models with self-rated health adjustment, the possibility that self-rated health mediates part of the CLS-pain relationship cannot be fully resolved in this study design. Fifth, pain was assessed through a single self-report item (“Are you often troubled with pain?”), which does not distinguish between pain locations or whether pain met formal criteria for chronic pain classification; this limits the precision of the pain incidence outcome. Finally, although the longitudinal design allows for temporal ordering of stressors and pain onset, the 2-year observation window between HRS waves precludes conclusions about causality and does not capture stressor or pain fluctuations occurring within that interval.

## 5 Conclusion

This study provides longitudinal evidence that chronic life stressors—particularly self-health problems and cumulative stressor burden—significantly predict pain incidence in adults 50 and older, with each additional moderate-to-severe stressor increasing the odds of new-onset pain by 13%. These findings underscore the public health significance of modifiable psychosocial risk factors in pain development and support the integration of social needs screening into both clinical and community-based settings as a key pain prevention strategy among aging adults.

## Data Availability

Publicly available datasets were analyzed in this study. The Health and Retirement Study data used in this analysis are publicly available through the University of Michigan HRS data portal (https://hrs.isr.umich.edu/data-products).
